# Sexually dimorphic outcomes and inflammatory responses in hypoxic-ischemic encephalopathy

**DOI:** 10.1186/s12974-015-0251-6

**Published:** 2015-02-20

**Authors:** Mehwish A Mirza, Rodney Ritzel, Yan Xu, Louise D McCullough, Fudong Liu

**Affiliations:** Department of Neuroscience, University of Connecticut Health Center, 263 Farmington Avenue, Farmington, CT 06030 USA; Department of Neurology, University of Connecticut Health Center, 263 Farmington Avenue, Farmington, CT 06030 USA

**Keywords:** Hypoxic-ischemic encephalopathy, Neonate, Inflammation, Infarct, Microglia

## Abstract

**Background:**

Neonatal hypoxic-ischemic encephalopathy (HIE) is an important cause of motor and cognitive impairment in children. Clinically, male infants are more vulnerable to ischemic insults and suffer more long-term deficits than females; however, the mechanisms underlying this sex difference remain elusive. Inflammatory processes initiated by microglial activation are fundamental in the pathophysiology of ischemia. Recent studies report a sexual dimorphism in microglia numbers and expression of activation markers in the neonatal brain under normal conditions. How these basal sex differences in microglia affect HIE remains largely unexplored. This study investigated sex differences in ischemic outcomes and inflammation triggered by HIE. We hypothesize that ischemia induces sex-specific brain injury in male and female neonates and that microglial activation and inflammatory responses play an important role in this sexual dimorphism.

**Methods:**

Male and female C57BL6 mice were subjected to 60-min Rice-Vanucci modeling (RVM) at post-natal day 10 (P10) to induce HIE. Stroke outcomes were measured 1, 3, 7, and 30 days after stroke. Microglial activation and inflammatory responses were evaluated by flow cytometry and cytokine analysis.

**Results:**

On day 1 of HIE, no difference in infarct volumes or seizure scores was seen between male and female neonates. However, female neonates exhibited significantly smaller infarct size and fewer seizures compared to males 3 days after HIE. Females also had less brain tissue loss and behavioral deficits compared to males at the chronic stage of HIE. Male animals demonstrated increased microglial activation and up-regulated inflammatory response compared to females at day 3.

**Conclusions:**

HIE leads to an equivalent primary brain injury in male and female neonates at the acute stage that develops into sexually dimorphic outcomes at later time points. An innate immune response secondary to the primary injury may contribute to sexual dimorphism in HIE.

## Background

Hypoxic-ischemic encephalopathy (HIE) is a common cause of long-term neurological sequelae and hemiplegic cerebral palsy in children [1]. Male sex is a risk factor for neonatal HIE [2]. Clinical evidence demonstrates that among infants at risk for HIE, females are at a quite significant advantage over males. Males are two times more likely to experience prenatal anoxia, hemorrhage, and infection and 1.8 times more likely to suffer cerebral birth trauma suggesting sex differences in the incidence of HIE [3-5]. Overall, childhood ischemic injury appears to be more common in boys regardless of age, stroke subtype, or history of trauma [1,6]. Notably, boys also experience worse recovery once an injury occurs. Male infants suffer more long-term cognitive deficits compared to their female counterparts with comparable hypoxic-ischemic injury [7]. The mechanisms underlying this sexual dimorphism in HIE remain elusive. It is well established that estrogen plays a neuroprotective role in adult ischemic stroke [8]. However, hormones may not play a significant role in the HIE sexual dimorphism as hormone levels are equivalently low between male and female neonates [9].

Cerebral ischemia induces both a central and a peripheral inflammatory response which contributes to secondary neuronal damage [10]. The immune response is primarily initiated by activation of microglia, the major resident immune cells in the brain [11,12]. Once activated, microglia develop macrophage-like capabilities including phagocytosis, cytokine production, antigen presentation, and the release of matrix metalloproteinases (MMPs) that disrupt the blood brain barrier (BBB) [13]. As a result, peripheral leukocytes infiltrate into the brain and the normally immune-privileged brain environment is exposed to systemic responses that further exacerbate inflammation and brain damage. Recent studies report a sexual dimorphism in microglia numbers and expression of activation markers in neonatal brains under normal conditions [14-16]. How these basal sex differences in microglia affect stroke phenotypes and inflammation triggered by HIE remains largely unexplored. In this study, we utilized the Rice-Vanucci model (RVM) in male and female wild-type littermate mice to induce HIE at post-natal day 10 (P10) to investigate the sex difference in immune responses and HIE outcomes.

## Methods

### Experimental animals

Wild-type C57BL/6 P10 mice were utilized to model HIE. This study was conducted in accordance with National Institutes of Health guidelines for the care and use of animals in research and under protocols approved by the Animal Care and Use Committee of the University of Connecticut Health Center.

### RVM model

The RVM model was modified for use in mice [17,18]. Briefly, P10 mice were anesthetized with 1.5% isoflurane and placed in the supine position. Body temperature was maintained at 36.5°C with an automated temperature control feedback system. The fur and skin of the frontal neck region were disinfected with 70% ethanol, and a 5-mm midline incision was made to expose the right common carotid artery (CCA). Double permanent ligation of CCA was made with a 6–0 silk suture, and the incision was then closed and the pups were returned to their dams. Sham mice underwent the same procedure except ligation of the right CCA. One hour after surgery, pups were placed in a 36°C chamber containing 10% oxygen and 90% nitrogen for 60 min to induce ischemic injury as in [19]. Mice were sacrificed for 1-, 3-, 7-, or 30-day survival after stroke.

### Behavioral testing

Seizure activity was scored according to a seizure rating scale as previously reported [20-23] (*n* = 6/group). Every 5 min in 1 h at the same time of 1 day and 3 days after HIE, the score corresponding to the highest level of seizure activity observed during that time period was recorded and summed to produce a total seizure score. Seizure behavior was scored as follows: 0 = normal behavior; 1 = immobility; 2 = rigid posture; 3 = repetitive scratching, circling, or head bobbing; 4 = forelimb clonus, rearing, and falling; 5 = mice that exhibited level four behaviors repeatedly; and 6 = severe tonic-clonic behavior. The corner test was performed to assess for forelimb asymmetry at 7 and 30 days of HIE as previously described [19,24] (*n* = 9 for males; *n* = 10 for females). The mouse was placed between two cardboard pieces (size of each: 30 × 20 × 1 cm). The two boards were gradually moved to close the mouse from both sides to encourage the mouse to enter into a corner of 30° with a small opening along the joint between the two boards. When the mouse entered the deep part of the corner, both sides of the vibrissae were stimulated together by the two boards. Then, the mouse reared forward and upward, and then turned back to face the open end. Twenty trials were performed for each mouse and the percentage of right turns was calculated. Only turns involving full rearing along either board were recorded.

### Histological assessment

At the indicated time points, mice were euthanized and the brains were removed and cut into five 2-mm slices. The slices were stained with 1.5% 2,3,4-triphenyltetrazolium chloride (TTC) solution at 37°C for 30 min and fixed with 4% formalin [25] (*n* = 9 for males; *n* = 10 for females). Separate cohorts of HIE brains were perfused and stained with cresyl violet (CV) for measurement of chronic tissue loss as previously described [19,26] (*n* = 6/sex). Images were digitalized and brain infarct volumes (% contralateral hemisphere structure, corrected for edema) were analyzed using computer software (Sigmascan Pro5) as previously described [25,26].

### Flow cytometry

Leukocytes from brain tissue were prepared as previously described [27,28]. Animals (*n* = 6/sex for HIE; *n* = 3/sex for sham) were anesthetized with Avertin (2, 2, 2-tribromoethanol) and intracardially perfused with phosphate-buffered saline (PBS) for 5 min. Brains were harvested and dissected to isolate the ipsilateral stroke/sham hemisphere. The dissected brains were placed in Roswell Park Memorial Institute (RPMI) 1640 complete medium (10% fetal calf serum, 1% sodium pyruvate, 1% non-essential amino acid, 0.1% β mercaptoethanol, 100 U/ml of penicillin, and 100 μg/ml of streptomycin; Sigma-Aldrich, St. Louis, MO, USA) in separate tubes on ice. The brains were mechanically dissociated and incubated with 100 μl of collagenase/dispase (1 mg/ml, Roche Diagnostics, Indianapolis, IN, USA) and 300 μl DNAse I (10 mg/ml, Roche) for 45 min at 37°C. After incubation, the brain homogenate was passed through 1 ml pipette tip several times and harvested in 20 ml complete RPMI. The cells were pelleted at 1,200 G, 4°C for 10 min, re-suspended in 40 ml complete RPMI, passed over a 70 μm cell strainer and pelleted again. The filtered cells were re-suspended in a 70%/30% Percoll gradient (GE Healthcare, Pewaukee, WI, USA) and spun at 2,000 rpm for 25 min at room temperature with no brake. Myelin was removed from the top and cells collected from the interface into 12 ml complete RPMI. The cells were washed and re-suspended in 300 ml FACS buffer for antibody staining and counting. Fluorophore-conjugated antibodies against CD45 (#8017-9459), CD11b (#56-0112), Ly6G (#48-5931), Ly6C (# 45–5932), and MHCII (#11-5321) were obtained from eBioscience.

### ELISA

Blood was extracted and centrifuged at 6,000 rpm for 10 min at 4°C (*n* = 12/sex for HIE; *n* = 3/sex for sham). The supernatant was collected to isolate serum from the blood. Hormone levels were measured using ELISA kits for testosterone (#TE187S-100; Calbiotech, Spring Valley, CA, USA) and estrogen (#BQ180S; BQ Kits, San Diego, 122 CA, USA). The serum concentration of TNF-α and IL-1β was determined by mouse TNF-a (#88-7324) and IL-1β (#88-7013) ELISA kits (eBioscience).

### Statistics

Data from individual experiments were presented as mean ± SEM and analyzed with a *t*-test (infarct volumes, hormone levels, and corner test scores) or two-way ANOVA (seizure scores, cytokines, and flow cytometry data). *P* < 0.05 was considered statistically significant. Investigators were blinded to mouse sex for stroke surgery, behavioral testing, infarct, and inflammation analysis.

## Results

### Male neonates had worse histological damage after HIE than females

**Figure 1:**
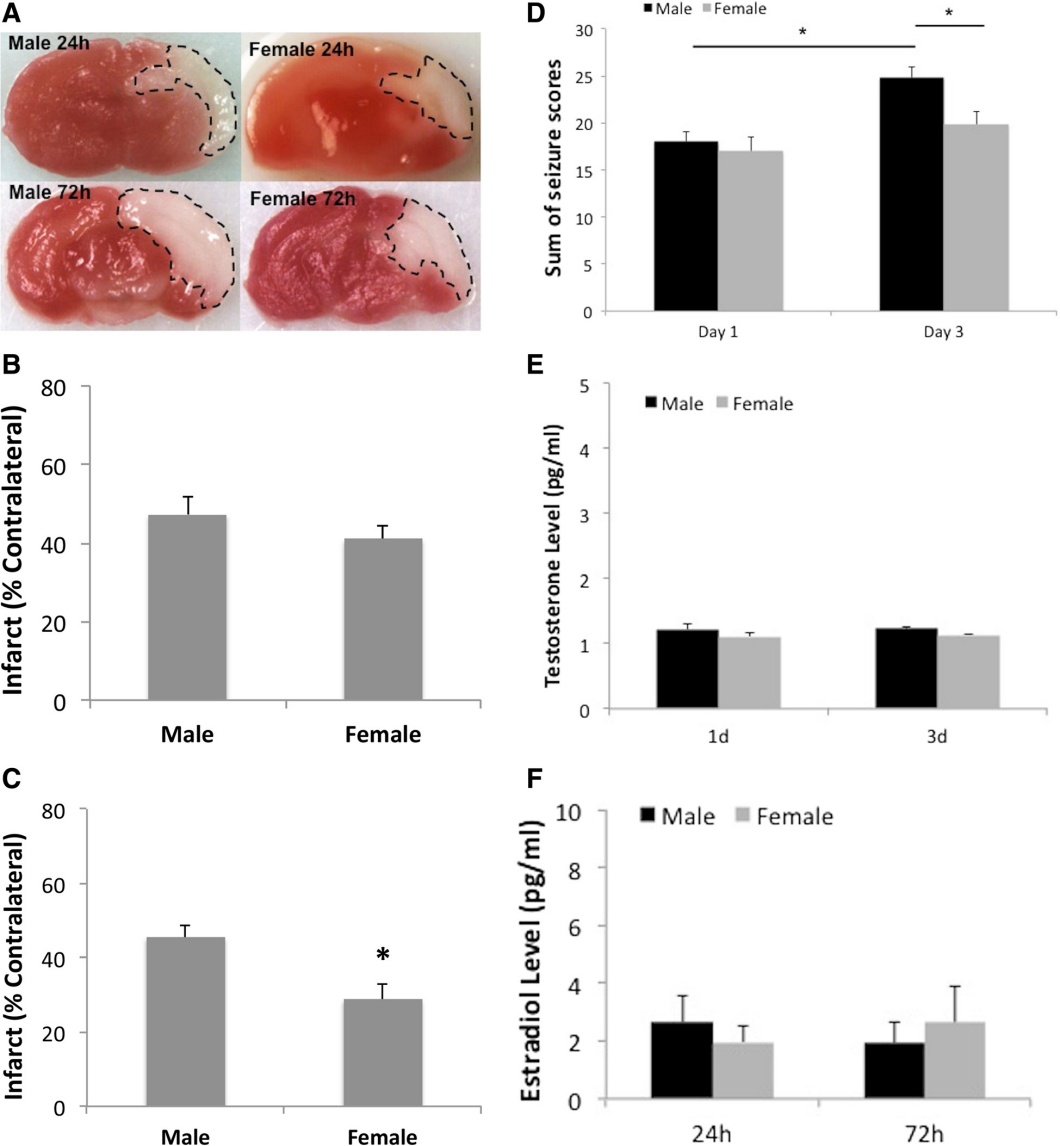
**Acute stroke outcomes at 1 day and 3 days after HIE. (A)** Representative coronal brain sections of TTC-stained brains at 1 day and 3 days. The black dotted line outlines the border of the infarct; red-colored area: unaffected tissue; white-colored area: infarcted tissue. **(B, C)** Quantification of infarct volumes at 1 day **(B)** and 3 days **(C)** of HIE. *P < .05 vs. male. **(D)** Seizure scores at 1 day and 3 days of HIE. *P < .05. **(E, F)** Testosterone **(E)** and estradiol **(F)** levels in serum.

We first examined HIE outcomes in P10 neonatal mice with RVM model. One day after HIE, male and female exhibited equivalent histological changes in the brain; however, male pups had significantly larger infarct in the ipsilateral hemisphere compared to females at 3 days of HIE (Figure 1A, B, C). Correspondingly, male pups had higher seizure scores than females 3 days after HIE, whereas no differences were seen at 1 day (Figure 1D). To investigate whether differences in hormone levels could contribute to HIE outcomes, we measured serum levels of testosterone and estradiol at terminal endpoints. No significant differences in either testosterone (Figure 1E) or estradiol (Figure 1F) levels were seen between males and females at either time point, suggesting factors other than hormones are responsible for the differing HIE outcomes in male vs. female neonates.

### Serum cytokine levels differ between male and female mice after HIE

**Figure 2:**
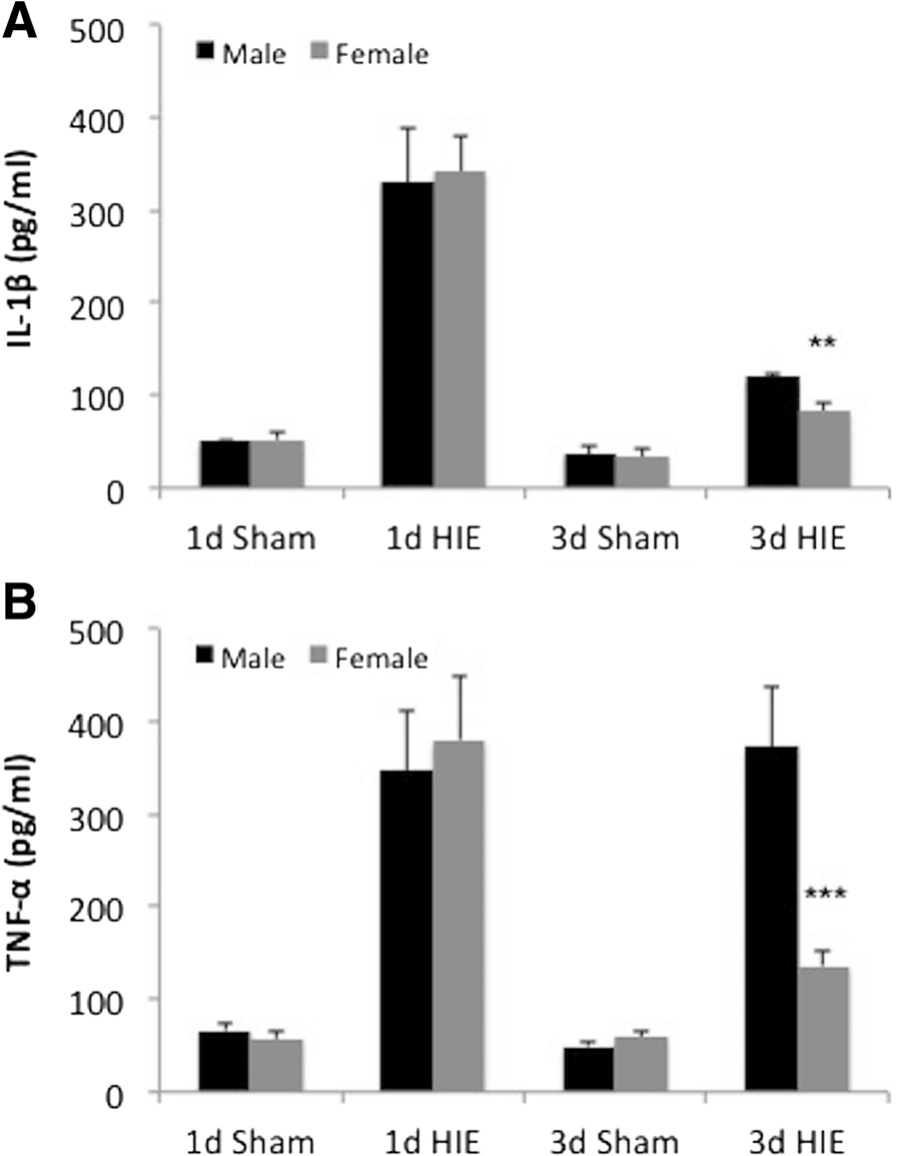
**Cytokine levels in serum. (A)** IL-1β and **(B)** TNF-α levels are significantly higher in males than in females 3 days after HIE. **P < .01 vs. 3-day males; ***P < .001 vs. 3-day males.

The innate immune response to ischemia induces secondary brain injury following acute ischemic damage [29]. To investigate whether sex differences in inflammatory responses contribute to the delayed sexual dimorphism in HIE outcomes, we measured IL-1β, TNF-α, and IL-6 levels in the serum of the mice in the infarct cohort. Interestingly, the results of IL-1β and TNF-α were consistent with the infarct data: although there were no sex differences in IL-1β and TNF-α levels at 1 day, males had significantly higher serum concentration of both cytokines compared to females 3 days after HIE (Figure 2A, B). *No sex differences in IL-6 levels were seen at either 1 day or 3 days of HIE (data not shown)*.

### MHC II was differentially expressed on microglia in male vs. female ischemic brain

**Figure 3:**
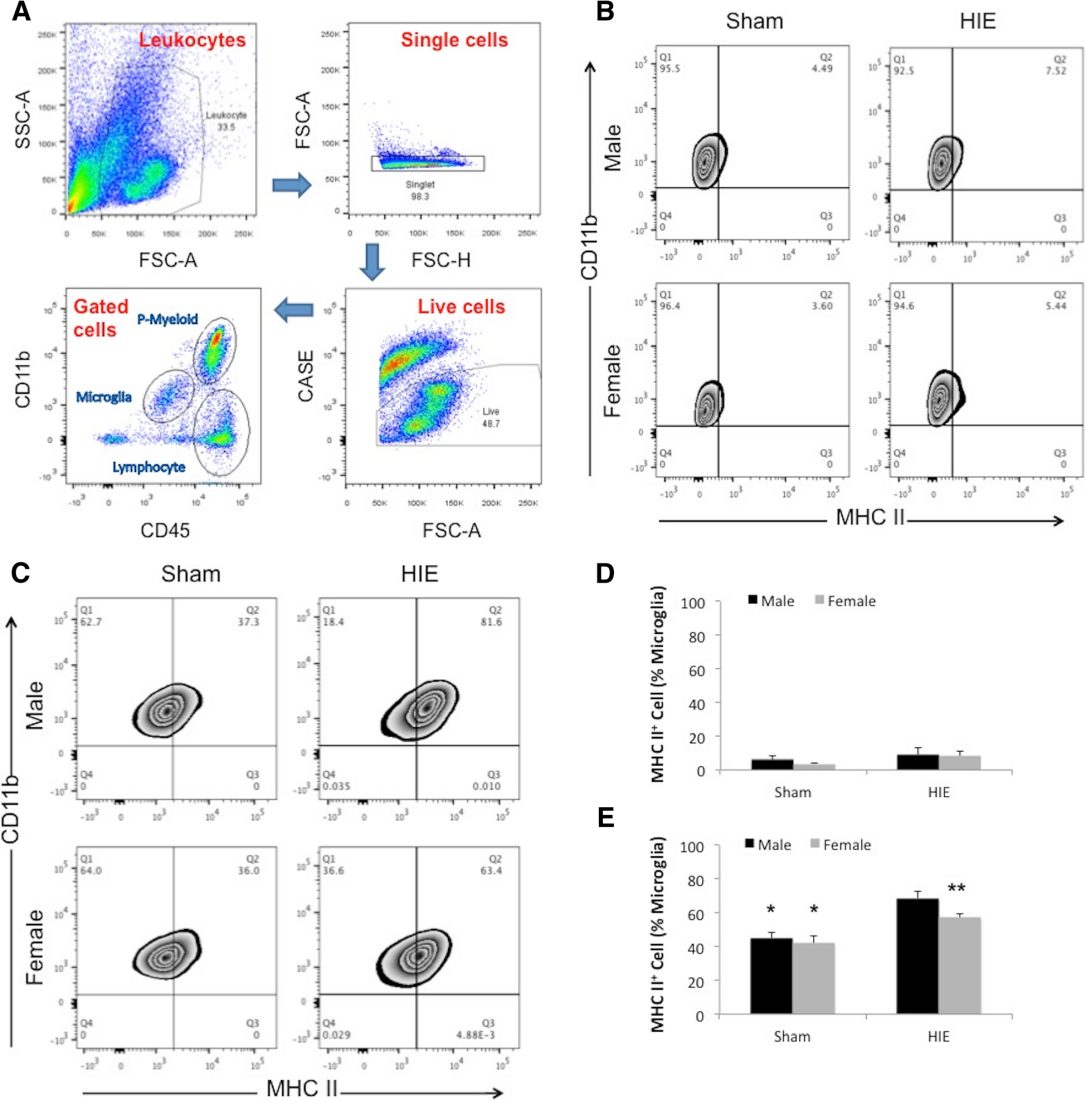
**Microglial activation after HIE. (A)** Gating strategy used to isolate peripheral myeloid cells, microglia, and lymphocytes. **(B, C)** Analysis of gated microglia at 1 day **(B)** and 3 days **(C)** of HIE: the upper right quadrant indicates MHC II^+^ microglia. **(D, E)** Quantification of MHC II^+^ microglia percentage of total microglia at 1 day **(D)** and 3 days **(E)** of HIE. *P < .05 vs. HIE counterpart; **P < .05 vs. HIE males.

We next examined microglial activation after HIE with flow cytometry as microglial activation is a key element in initiating and perpetuating inflammatory responses to ischemia [30,31]. MHC II is a well-established marker of microglial activation [32,33]. We quantified the percentage of MHC II^+^ microglia to total microglia using flow cytometry. The gating strategy is shown in Figure 3A (CD45^low^CD11b^+^ for microglia). As shown in Figure 3B, D, no difference in the percentage of MHC II^+^/total microglia (upper right quadrant in each flow plot) was seen between sham and stroke or between males and females at 1 day of HIE. However, both males and females had higher percentage than their corresponding shams at 3 days; in addition, the percentage was significantly higher in males vs. females in HIE groups (Figure 3E).

### Infiltrating leukocyte recruitment was higher in male vs. female ischemic brain

**Figure 4:**
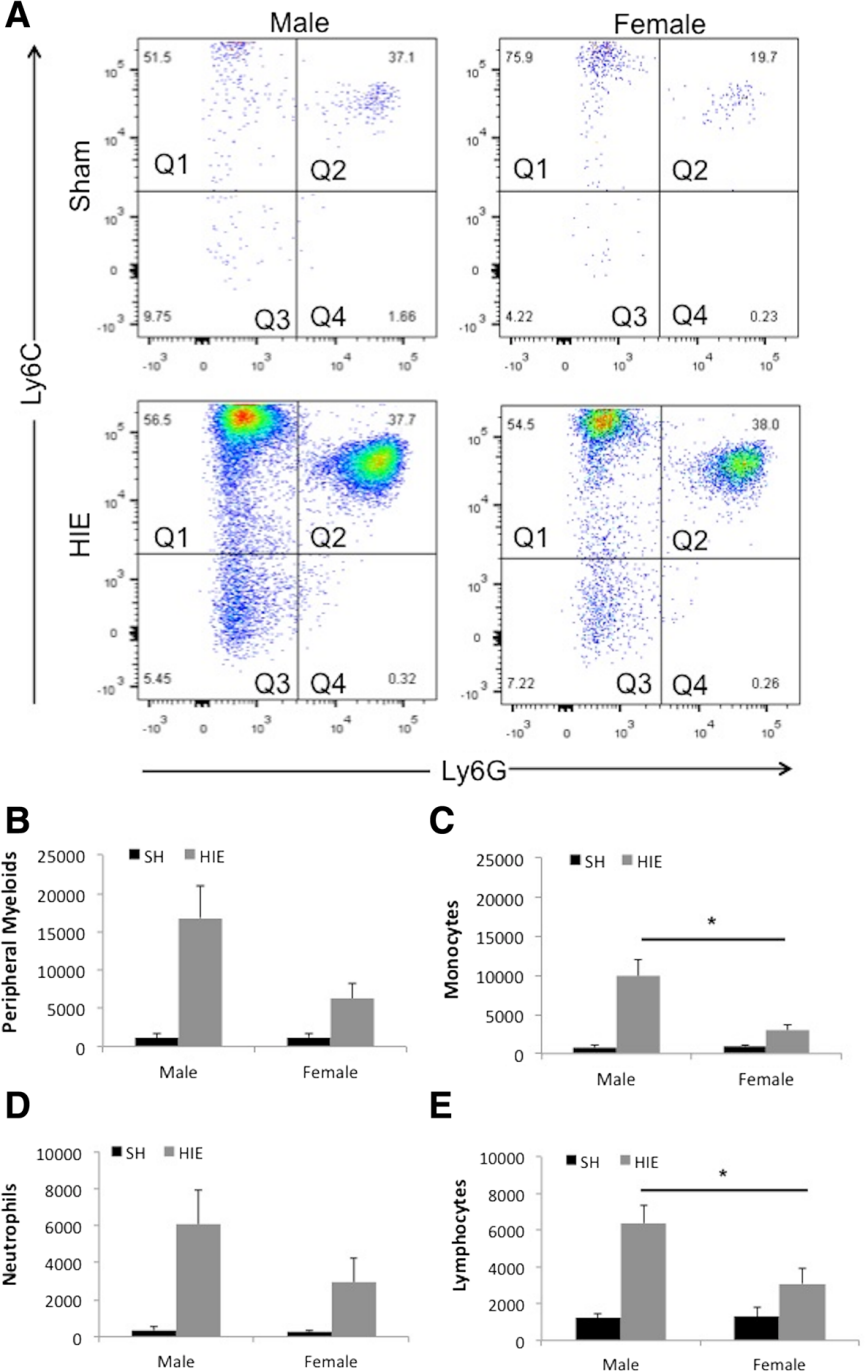
**Infiltration of peripheral leukocytes in the ipsilateral hemispheres at 3 days of HIE. (A)** Analysis of gated peripheral myeloid cells. Q1 quadrant: monocytes; Q2 quadrant: neutrophils. **(B, C, D, E)** Quantification of total peripheral myeloid cells, monocytes, neutrophils, and lymphocytes in HIE brains. *P < .05.

Inflammatory responses involve activation of resident immune cells (microglia) and infiltration of peripheral leukocytes in the ischemic brain. To examine the infiltrating leukocytes with flow cytometry, we gated monocytes as CD45^high^CD11b^+^Ly6C^+^Ly6G^−^, neutrophils as CD45^high^CD11b^+^Ly6G^+^, total peripheral myeloid cells as CD45^high^CD11b^+^, and lymphocytes as CD45^high^CD11b^−^ (Figures 3A and 4A). There were significantly more monocyte and lymphocyte infiltration in male vs. female brains 3 days after HIE (Figure 4A, C, E). Peripheral total myeloid cells and neutrophils also showed a trend towards increased levels in male brains, but this did not reach significance (Figure 4A, B, D). No sex differences in infiltration of peripheral leukocytes were seen at 1 day of HIE (data not shown).

### Female mice had less behavioral deficits compared to males in the chronic phase of HIE

**Figure 5:**
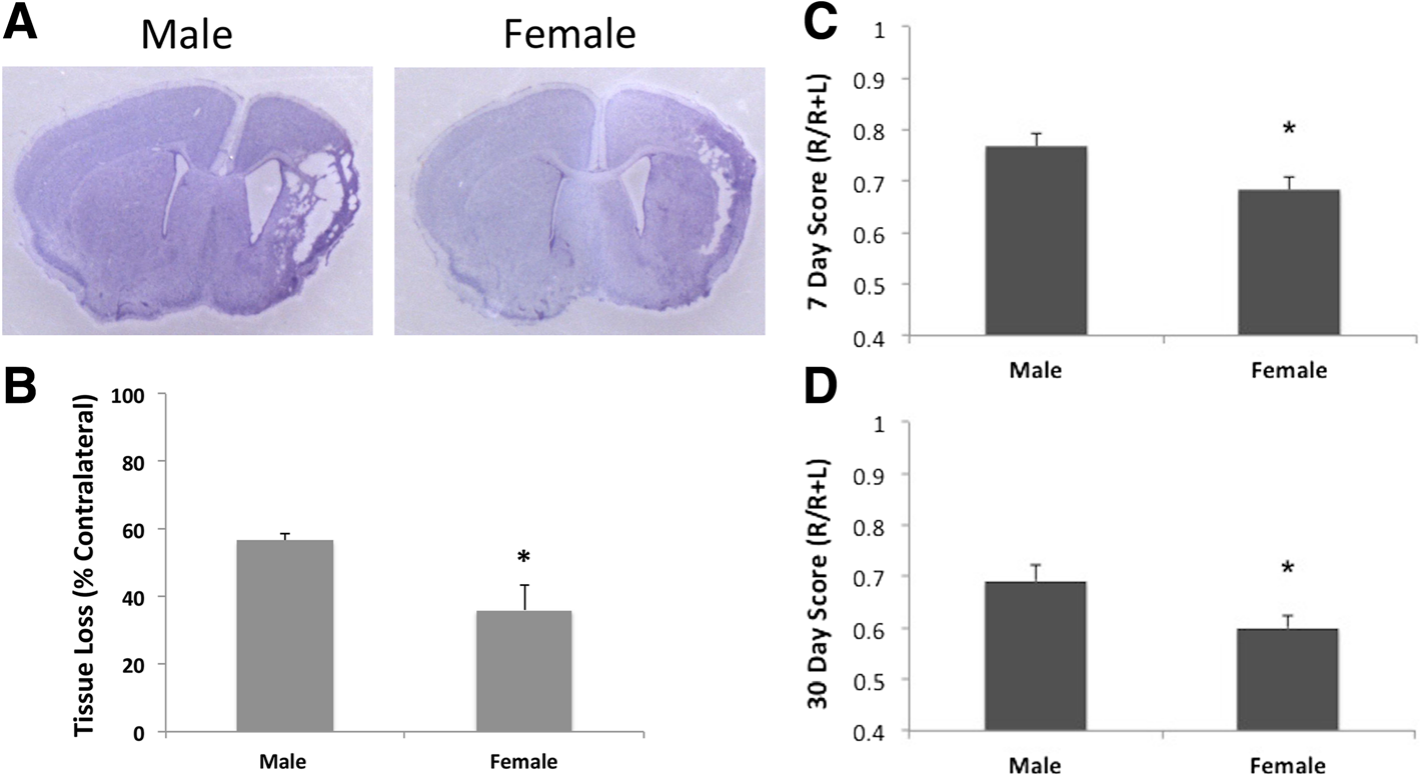
**Chronic HIE outcomes.** **(A)** Representative images of brain slices stained with cresyl violet (CV) 30 days after HIE. Note that the cavitation and atrophy can be seen in both the male and female brains. **(B)** Quantification of brain tissue loss at 30 days of HIE. *P < .05 vs. males. **(C, D)** Quantification of corner test scores at 7 days **(B)** and 30 days **(C)** of HIE. *P < .05 vs. males.

We also examined outcomes at 7 days and 30 days after HIE; the infarct becomes less visible at the chronic stages and the ischemic brains exhibit either cavitation or atrophy due to the tissue loss (Figure 5A). Tissue loss was measured and quantified at the endpoint (30 days), and male animals had significantly more tissue loss than females (Figure 5B). We performed behavioral studies on HIE mice at the chronic stages to evaluate long-term outcomes. Female mice had significantly less forelimb asymmetry vs. males at both 7 days and 30 days after HIE (Figure 5C, D).

## Discussion

Sexual dimorphism in HIE has long been recognized clinically; nevertheless, the underlying mechanism remains elusive. The present study employed a widely utilized HIE model (RVM) to study these sex differences and revealed several important new findings. Firstly, hypoxic-ischemic insults lead to an equivalent primary injury in male and female neonatal brains. However, this ischemic damage evolves differently in each sex with males showing significantly worse histological damage at later time points. Circulating hormone levels are not responsible for this sex difference, as the serum levels of testosterone and estradiol were equivalent in males and females. Secondly, HIE induces microglial activation in a time-dependent manner, as microglia were significantly activated at 3 days but not at 1 day after HIE. Thirdly, consistent with HIE outcomes, a delayed sex difference in both central and peripheral immune responses exists evidenced by activation of microglia, infiltration of peripheral leukocytes, and expression of inflammatory cytokines (Figures 2, 3, and 4). Finally, the sex difference in neonatal HIE outcomes was not attenuated by development as these difference extended to the chronic stages of HIE.

Cerebral ischemia is a sexually dimorphic disease throughout the life span [34]; however, the underlying mechanisms responsible for these differences may differ with age. Sexual dimorphism in adult stroke has been largely attributed to the protective effects of estrogen [35,36]. In children, estradiol levels are equivalently low in males and females until adolescence [37]. Male neonatal mammals (rat, mouse, horse, human, etc.) undergo a testosterone surge within 24 h of birth, and then the levels decrease and approaches that of females during the remainder of the neonatal period [38]. Consistent with previous studies, our data showed that serum testosterone and estradiol levels are equivalent between males and females, suggesting that circulating gonadal hormones do not mediate the sexually dimorphic phenotype of HI-induced neonatal brain damage. The testosterone surge in newborn males figures prominently in the development of mechanisms controlling gonadotropin, sexual behavior, and also promotes the functional differentiation of the accessory sex glands [38]. Whether the sex difference in HIE is related to the early organizational effects of hormones remains unknown. However, in our study, the HI insult did not induce a sex difference in outcomes until 3 days after HIE (Figure 1), suggesting a differential down-stream mechanistic signaling in males vs. females may exist secondary that is triggered by the primary HI damage. Of note, the principal form of hypoxia-ischemic brain injury in the immature brain involves cerebral white matter [39]. The hypoxic-ischemic lesion induced by RVM in the present study includes both the white and gray matter which cannot be distinguished with TTC staining. Therefore, the sex differences revealed in the study reflect morphological and functional changes after HIE in the whole brain instead of the white matter only.

Brain ischemia is a powerful stimulus that triggers a series of events that lead to the rapid activation of resident microglia as well as mobilization and infiltration of circulating leukocytes [30,40] to elicit a secondary neuronal damage. This process is modulated by several cell adhesion molecules and cytokines which, when induced, act upon the vascular endothelium to increase the expression of ICAM-1, P-selectin, and E-selectin, leading to further local accumulation and adhesion of leukocytes [41-43]. After gaining entry into the central nervous system (CNS) through the BBB, infiltrating leukocytes release cytokines and chemokines, amplifying the intrinsic (microglial) brain inflammatory response further over the next few days. Microglial activation and aggregation is a pathological marker for HIE in human infants [44]. Retrospective clinical studies on postmortem examinations from neonatal brains found patients who died of HIE had a dense infiltrate of microglia in the hippocampal dentate gyrus, whereas those dying of other acute causes (trauma or sepsis) had significantly fewer microglia [44]. The present study revealed that the level of microglial MHC II, a widely used marker of microglial activation, was not significantly increased until 3 days after HIE, suggesting the pro-inflammatory response is delayed in neonatal ischemic brains and therefore may be an attractive target for therapeutic intervention. However, microglia are not equally activated in males vs. females, as males had significantly more MHC II^+^ cells than females at 3 days of HIE. Interestingly, previous studies reported that neonatal microglia exhibit sex-specific profiles even under normal conditions. For example, neonatal male mice had twice as many microglia as female mice in the preoptic area; microglial inhibition during the critical period for sexual differentiation prevented sex differences in microglia and adult copulatory behavior [14]. In our model, we did not observe a sex difference in microglial activation at the baseline in the whole hemisphere (Figure 3D, E); however, it is likely that male microglia are primed towards activation and are more activated once an ischemic event occurs. MHC II is increasingly used as a marker of M1 microglial activation (classical activation) that is pro-inflammatory [30]. It was found that microglia are both M1 and M2 (alternative activation; anti-inflammatory) during the acute stage of neuroinflammation induced by adult stroke [45-47]; however, the M2 phenotype is transient and soon transitions to a skewed M1 that persists up to 2 weeks after stroke [46]. Therefore, the significant difference in M1 phenotype of microglia in the present study may be critically related to the sexual dimorphism seen in HIE outcomes.

In addition to the sex difference in the activation of central immune cells (microglia), peripheral immune responses also exhibited a sex-specific profile, shown by the serum levels of IL-1β and TNF-α (Figure 2), and infiltration of peripheral leukocytes (Figure 4). IL-1β, TNF-α, and IL-6 are known to be the major inflammatory mediators with increased levels in HIE [48-50]. These cytokines are released by peripheral monocytes and macrophages, as well as by astrocytes and microglia [51,52]. In the present study, both the central and peripheral immune responses showed the same pattern as seen by histological outcomes after HIE, i.e., sex-specific changes at a later time point (3 days), suggesting HIE induces an equivalent primary brain injury initially in males and females that evolves differently later due to the secondary neuronal damage exerted by the post-ischemic inflammation. Overwhelming data have demonstrated that inflammatory responses cause secondary neuronal damage following acute ischemic injury [40,53,54]. Differences between males and females can be identified at many levels of the immune response (reviewed in [55]). Research on sex differences in immunology has centered on two main influences: endocrinology (the effect of sex hormones) and genetics (the effect of the X chromosome). In adults, estrogen has been proven to suppress inflammatory responses after stroke [56]; however, chromosomal effects may be a more important factor in post-ischemic inflammation in neonates as hormone levels are equivalent in males and females. Nevertheless, the organizational effect of hormones cannot be excluded given the fact that males undergo a testosterone surge both during embryogenesis and early after birth [38,57]. This can be addressed by dissociation of the chromosomal effect from hormonal organizational effect with the ‘four core genotypes’ mouse model [58], which is an on-going project in this lab. The sexual dimorphism in acute HIE outcomes extends to chronic stages, suggesting that the post-ischemic secondary neuronal damage exert a profound impact on the development of brain function. This underlines the importance of acute inflammatory responses in the pathophysiology of HIE. Treatments aimed at the early innate immune response may improve the long-term functional disability caused by this devastating disease.

In summary, neonatal HIE leads to an equivalent level of primary hypoxic-ischemic damage in the male vs. female brains at day 1 after injury. A sex difference subsequently develops with the progression of the disease. Circulating hormones are not contributing factors to this sexual dimorphism, whereas a striking sex-specific innate immune response is closely correlated to HIE outcomes. Although the causative evidence is lacking and warrants further exploration, this study represents the initial step in targeting inflammatory responses as an effective therapeutic avenue for HIE and suggests that sex-specific strategies should be developed.

## Abbreviations

BBB: blood brain barrier
CCA: common carotid artery
CNS: central nervous system
HIE: hypoxic-ischemic encephalopathy
PBS: phosphate-buffered saline
RVM: Rice-Vanucci model
